# Endoscopic ultrasound-guided precise embolization for refractory abdominal arteriovenous malformation

**DOI:** 10.1055/a-2489-7981

**Published:** 2024-12-17

**Authors:** Fangfang Guo, Wei Huang, Lu Hao, Hongtan Chen

**Affiliations:** 1 71069 Department of Gastroenterology, Zhejiang University School of Medicine First Affiliated Hospital, Hangzhou, China

Arteriovenous malformations (AVMs) are abnormal fistulas between arteries and veins without an intervening capillary bed 1 , which may lead to a variety of symptoms as they progress 2 . The primary treatments, including surgical resection and transcatheter arterial embolization, still carry risks like bleeding and incomplete resection 3 4 . Here, we present a case of a refractory AVM treated with an endoscopic ultrasound (EUS)-guided devascularization technique.

A 38-year-old man was admitted with abdominal discomfort lasting one month. Contrast-enhanced computed tomography (CT) and digital subtraction angiography (DSA) revealed an abdominal AVM involving the common hepatic artery and the portal vein. The patient underwent DSA-guided transarterial embolization, and most of the AVM was occluded ( Fig. 1 ).

**Fig. 1:**
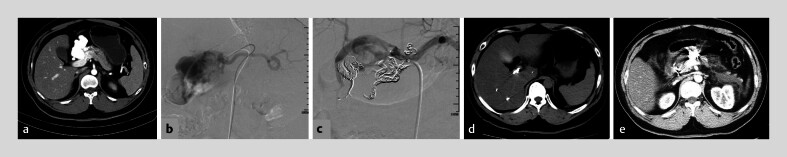
Abdominal computed tomography (CT) and digital subtraction angiography (DSA) images around the abdominal arteriovenous malformation. **a, b** Enhancement CT and DSA image showed the presence of arteriovenous malformations (AVMs) between the common hepatic artery and portal vein. **c** DSA image after interventional embolization. **d** CT scan showed the hepatic ectopic embolism after transarterial embolization. **e** CT scan showed multiple effusions around the pancreas and residual arteriovenous fistulas.

However, on the second day post-embolization, he developed severe abdominal pain, and an emergency CT indicated acute pancreatitis and a hepatic ectopic embolism. Despite standardized treatment, he experienced recurrent pain and significant weight loss. Follow-up CT and EUS identified an 8.5 × 6.8-cm pseudocyst in the pancreatic head and an approximately 4.0-cm residual AVM ( Fig. 2 ). To alleviate symptoms, we performed EUS-guided aspiration of the pseudocyst and placed a pancreatic duct stent.

**Fig. 2:**
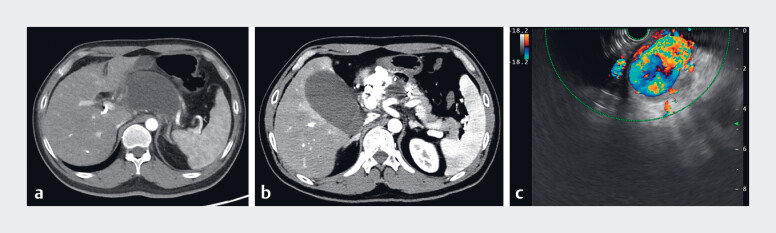
CT and endoscopic ultrasound (EUS) scan revealed the pseudocyst and residual arteriovenous fistula. **a** CT scan revealed a pseudocyst located in the pancreatic head. **b** Enhanced CT showed the residual arteriovenous fistula near the pancreatic head. **c** EUS with color Doppler revealed the inflow of the arteriovenous fistula.

Given the increasing size of the residual fistula and the complications from previous interventions, we opted for EUS-guided embolization of the residual AVM ( Video 1 ). Using a 22-gauge needle, we punctured the fistula cavity and sequentially deployed two coils (Boston Scientific, Marlborough, Massachusetts, USA), followed by consecutive injections of lauromacrogol and Histoacryl. EUS confirmed hyperechoic filling and cessation of inflow. Post-procedure, the patient reported no abdominal pain or fever and was discharged successfully. Follow-up EUS with color Doppler confirmed complete occlusion of the arteriovenous fistula ( Fig. 3 ).

Endoscopic ultrasound-guided embolization of the residual arteriovenous malformation.Video 1

**Fig. 3:**
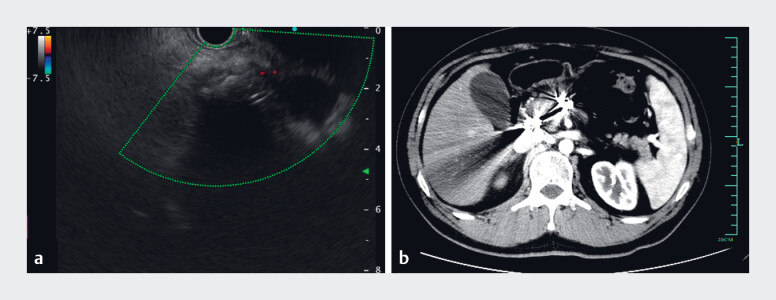
EUS and CT images at the 6-month follow-up after EUS-guided embolization. **a** EUS with color Doppler showed the complete occlusion of the arteriovenous fistula. **b** CT showed the complete occlusion of the arteriovenous fistula.

In conclusion, this case indicates the feasibility and efficacy of EUS-guided retrograde transvenous embolization for abdominal AVMs adjacent to the digestive tract. Moreover, EUS can be an ideal alternative for patient follow-up due to its real-time visualization of blood flow.

Endoscopy_UCTN_Code_TTT_1AS_2AL

## Notes

Endoscopy E-Videos https://eref.thieme.de/e-videos
*E-Videos* is an open access online section of the journal *Endoscopy* , reporting on interesting cases and new techniques in gastroenterological endoscopy. All papers include a high-quality video and are published with a Creative Commons CC-BY license. Endoscopy E-Videos qualify for HINARI discounts and waivers and eligibility is automatically checked during the submission process. We grant 100% waivers to articles whose corresponding authors are based in Group A countries and 50% waivers to those who are based in Group B countries as classified by Research4Life (see: https://www.research4life.org/access/eligibility/ ). This section has its own submission website at https://mc.manuscriptcentral.com/e-videos .
